# The intestinal tuft cell nanostructure in 3D

**DOI:** 10.1038/s41598-017-01520-x

**Published:** 2017-05-10

**Authors:** Ben Hoover, Valentina Baena, Melanie M. Kaelberer, Feven Getaneh, Skarleth Chinchilla, Diego V. Bohórquez

**Affiliations:** 1 School of Medicine, Duke University, Durham, NC USA; 2 Department of Cell Biology, University of Connecticut Health Center, Farmington, CT USA; 3 Department of Medicine, Duke University, Durham, NC USA; 4 Department of Neurobiology, Duke University, Durham, NC USA

## Abstract

Once referred to as “peculiar,” tuft cells are enigmatic epithelial cells. Here, we reasoned that future functional studies could be derived from a complete account of the tuft cell ultrastructure. We identified and documented the volumetric ultrastructure at nanometer resolution (4–5 nm/pixel) of specific intestinal tuft cells. The techniques used were Serial Block-Face (SBF) and Automated Tape-collecting Ultra-Microtome (ATUM) Scanning Electron Microscopy (SEM). Our results exposed a short (~15 µm) basal cytoplasmic process devoid of secretory vesicles. Volume rendering of serial sections unveiled several thin cytospinules (~1 µm). These cytospinules project from the tuft cell into the nuclei of neighboring epithelial cells. Volume rendering also revealed within the tuft cell an elegant network of interconnected tubules. The network forms a passage from the base of the microvilli to the rough endoplasmic reticulum. Based on their location and microanatomy, the tuft cells’ cytospinules, and tubular network, might facilitate the exchange of molecular cargo with nuclei of neighboring cells, and the gut lumen.

## Introduction

In 1956, Jarvi and Keyrilainen noted a cell type protruding into the lumen of the mouse stomach. They christened the cell as “the peculiar cell”^1^. Over the last 60 years, the name has evolved to ciliated^2^, brush^3^, and finally to tuft cell^4^ but its precise structure and function remains an enigma. The tuft cell is found throughout the body, in epithelial layers lining the salivary ducts^5^, trachea^6^, bronchi^7^, gall bladder^3^, bile ducts^8^, pancreas^9^, and the bowel^4^. Across tissues tuft cells are flask shaped with a microvillus tuft extending into the lumen. Some of the cells also have “lateral microvilli” but the purpose is unclear^10^. At the base of the microvilli tuft, there is a prominent cluster of vesicles and caveoli, which has been suggested to form a network but the evidence is inconclusive^11^. Since tuft cells were first reported, about a third of the published articles have focused on their ultrastructure and provided evidence of at least five peculiar features. These are the following:

1. Associated with the microvilli, there are some undefined spherical bodies, about 2 nm in diameter, known as glycocalceal bodies^12^.
2. Based on their close proximity to nerve fibers, tuft cells are thought to be innervated, nonetheless evidence of direct physical contact is absent^13^.
3. Tuft cells have a small basal cytoplasmic process, similar to neuropods in enteroendocrine cells, albeit devoid of secretory vesicles^14^.
4. Lateral cytoplasmic projections have been observed extending from the tuft cell body but their trajectory is unknown^10, 11^.
5. At the base of the cell’s microvilli, there is evidence of a cluster of vesicles and caveola. This molecular complex is referred to as a tubulovesicular network but the true continuity of the network has yet to be demonstrated^11^.

These features have been observed using classic Transmission Electron Microscopy (TEM). TEM has an unparalleled resolution in X and Y dimensions but the field of view is limited to only a few hundred square microns and automated access to the Z dimension is not possible. In the absence of the z dimension, only a fragmented view exists of the tuft cell at nanometer resolution. The invention of volumetric electron microscopy techniques, however, is spurring a renaissance in the study of cellular ultrastructure. Two methods are the most prominent: Serial Block Face Scanning Electron Microscopy (SBEM)^15^ and Automated Tape-collecting Ultra-Microtome (ATUM) Scanning Electron Microscopy^16^. Both techniques allow for the imaging of hundreds of serial sections, as thin as 30 nm, at a resolution as high as 4 nm/pixel. Although the resolution is still not comparable with traditional transmission electron microscopy, SBEM or ATUM can be used to resolve small organelles, such as a 30 nm synaptic vesicle^15, 17^. Stacked images can then be segmented using data visualization software, such as Imaris 8.3 (Bitplane), to render structures in three dimensions and reveal hidden ultrastructural details of the cells and their organelles. Here we used a correlative method^18^ to identify tuft cells in the ileum and colon and reveal their complete ultrastructure.

## Results and Discussion

### Identifying tuft cells for volumetric electron microscopy

**Figure 1:**
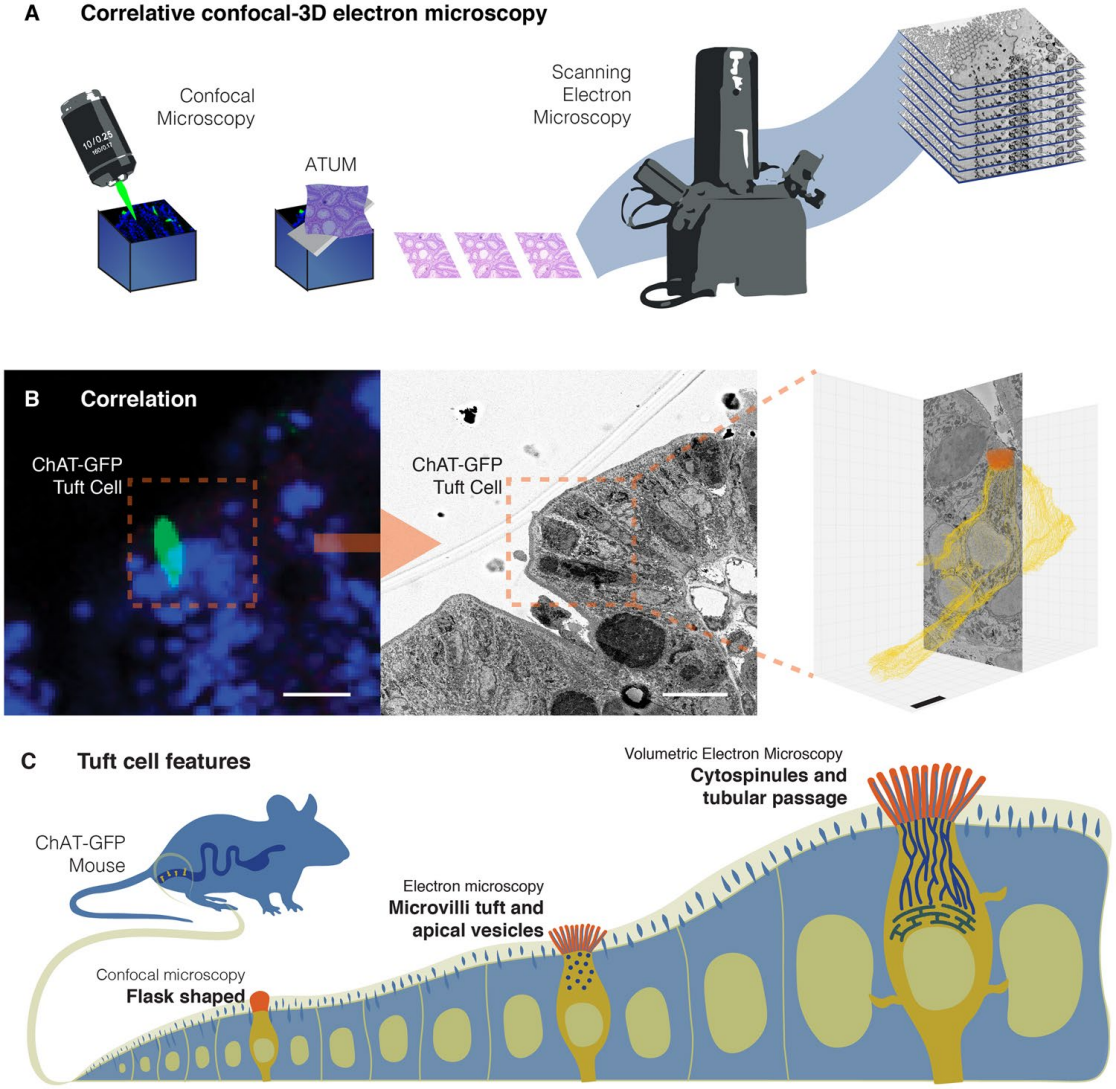
Identifying tuft cells for volumetric electron microscopy. (**A**) Overview of correlative method to identify a specific cell by fluorescence, then performing targeted scanning electron microscopy to uncover the ultrastructure of the desired cell in the third dimension. (**B)** A tuft cell in the colonic epithelium of ChAT-GFP transgenic mice is identified by fluorescence (B-left) and ATUM SEM (B-center), then volume rendered from serial images using data visualization software (B-right). (**C**) Volumetric EM analysis of tuft cells revealed cytospinules and a gut-to-endoplasmic reticulum passage. Bars in B-left and middle = 10 µm, bar in B-right = 1 µm.

Tuft cells stand out by their prominent tuft of microvilli in electron micrographs. Nonetheless, the odds of finding a tuft cell in an electron micrograph is rare. For every 1000 cells in the intestinal epithelium, only four cells are of the tuft type^19^. Our team faced a similar challenge not long ago while documenting the complete ultrastructure of intestinal enteroendocrine cells. Depending on their subtype, such as those secreting the anorectic peptide YY (PYY), the ratio of enteroendocrine cells to epithelial cells can be as low as 6 in 10,000^20^. To overcome this obstacle, we previously developed a protocol to correlate fluorescence microscopy with volumetric electron microscopy (Fig. 1A)^18^. A cell of interest is identified by fluorescence in a tissue block and this fluorescence micrograph serves as a reference to localize the same cell by SBEM or ATUM (Fig. 1B). Once identified, a region of interest is reconstructed in a series of consecutive high resolution SBEM or ATUM images to reveal ultrastructural details of the desired cell (Fig. 1C).

We used Choline acetyl transferase (ChAT) as a marker, which is one of only a handful of molecular markers to identify tuft cells^21^. We confirmed that ChAT serves to distinguish tuft cells from their chemosensory homologs the enteroendocrine cells by breeding a triple transgenic animal (ChAT-GFP::PyyCRE::tdTomato) that labels both tuft cells and enteroendocrine cells. In intestinal tissues from these mice, cholinergic nerve fibers and tuft cells fluoresce green and are distinguished with ease from enteroendocrine cells labeled by red fluorescence (S1 Fig. 1).

### The volumetric ultrastructure of tuft cells

**Figure 2:**
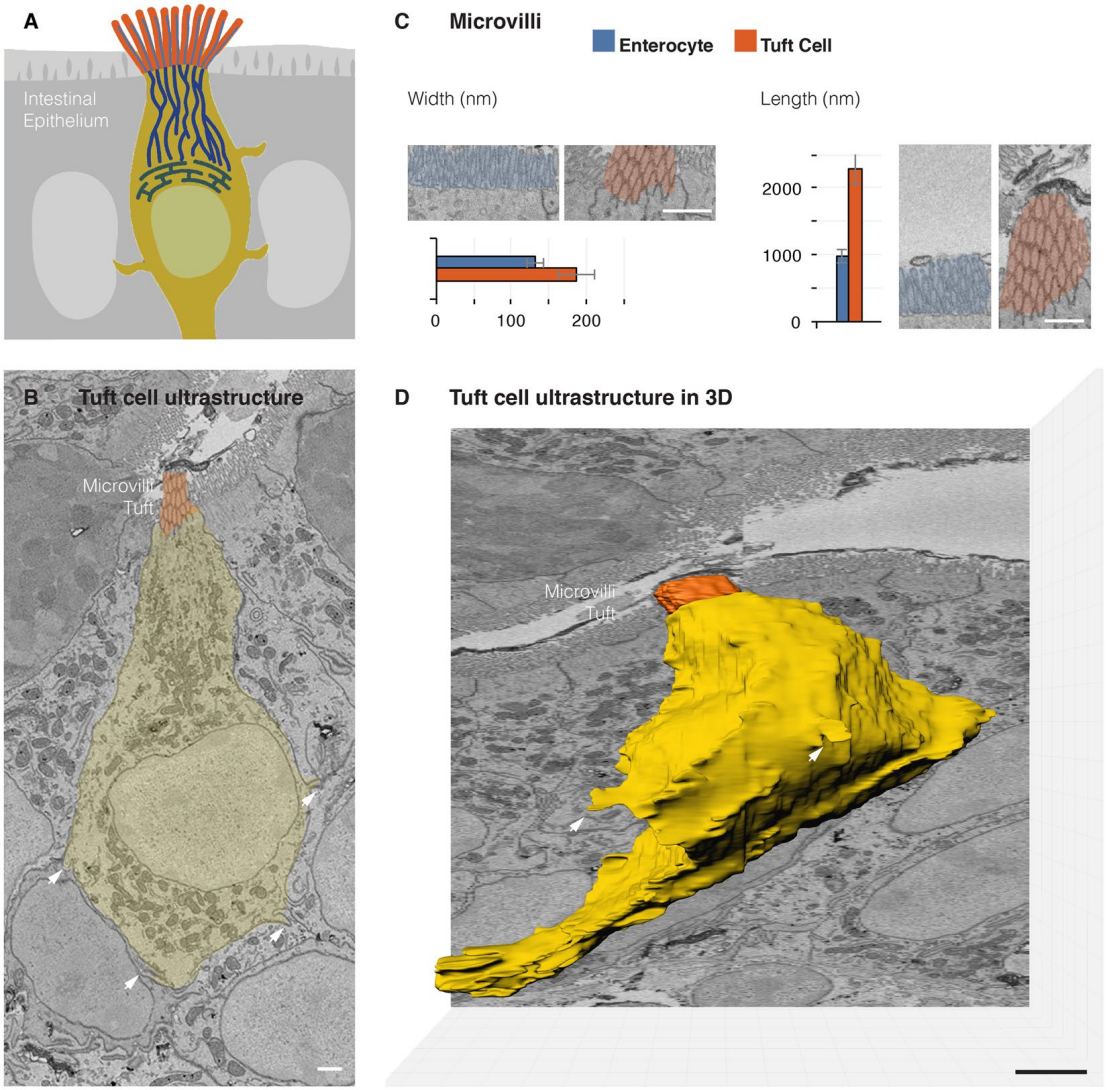
The ultrastructure of the tuft cell in the third dimension. (**A**) Schematic overview of the intestinal tuft cell. All data in panels are oriented from top-lumen to bottom-basal lamina. (**B**) An SBEM micrograph revealing a tuft cell in the mouse distal small intestine (yellow). Cytoplasmic spinules (arrows) projecting into adjacent cells and vesicles beneath the microvilli are evident at this resolution (5 nm/pixel). (**C**) Morphometrical assessment of tuft cell microvilli. Measurements are an average of 30 individual microvilli from three different cells in each group. (**D**) Volume rendering of 500 consecutive SBEM micrographs shows the complete 3D ultrastructure of tuft cell in the third dimension, including microvilli (orange), basal process, and cytospinules (arrows). See also Video 1. Bars = 1 µm.

Using the correlative method^18^, we identified a tuft cell in each of three different blocks of tissue from three different animals. Then, two blocks were analyzed using SBEM and one using ATUM. SBEM and ATUM, each, allow for automated serial sectioning and reconstruction of tissue ultrastructure in three dimensions but with one main difference. In SBEM, sectioning and imaging occur in sequence within the scanning electron microscope and the individual thin sections cannot be preserved for further analysis^15^. In ATUM, sectioning takes place outside of the scanning electron microscope prior to imaging but the thin sections can be preserved for further analysis^16^. The tissue blocks were about 1000 × 1000 × 50 µm. The identity of the chosen cells was confirmed in the SBEM and ATUM micrographs by its distinctive tuft of microvilli. Their microvilli are 0.187 µm (SD ± 0.024) wide and 2.289 µm (SD ± 0.222) long (Fig. 2B). By comparison, microvilli of adjacent enterocytes are 0.132 µm (SD ± 0.010) wide and 0.975 µm (SD ± 0.101) long. SBEM images were obtained at a resolution of 5 nm/pixel, sufficient to reveal lipid membranes and small secretory vesicles (Fig. 2C). The block was sectioned, and imaged, every 75 nm and the image stack was rendered by manual tracing using data visualization software Imaris Bitplane 3.0 to reveal the complete 3D ultrastructure of tuft cells (Fig. 2C). At the base of the cell, there was a single basal process extending away from the cell towards the basal lamina. The basal process in tuft cells resembled neuropods in enteroendocrine cells; though, unlike neuropods, it was devoid of secretory vesicles. No contact was observed between the tuft cells and underlying nerves. Also, extending from the cell body were several lateral and thorny projections penetrating deeply into neighboring epithelial cells. We referred to these as cytospinules (Video 1).

### Tuft cell cytospinules contact nuclei in neighbor cells

**Figure 3:**
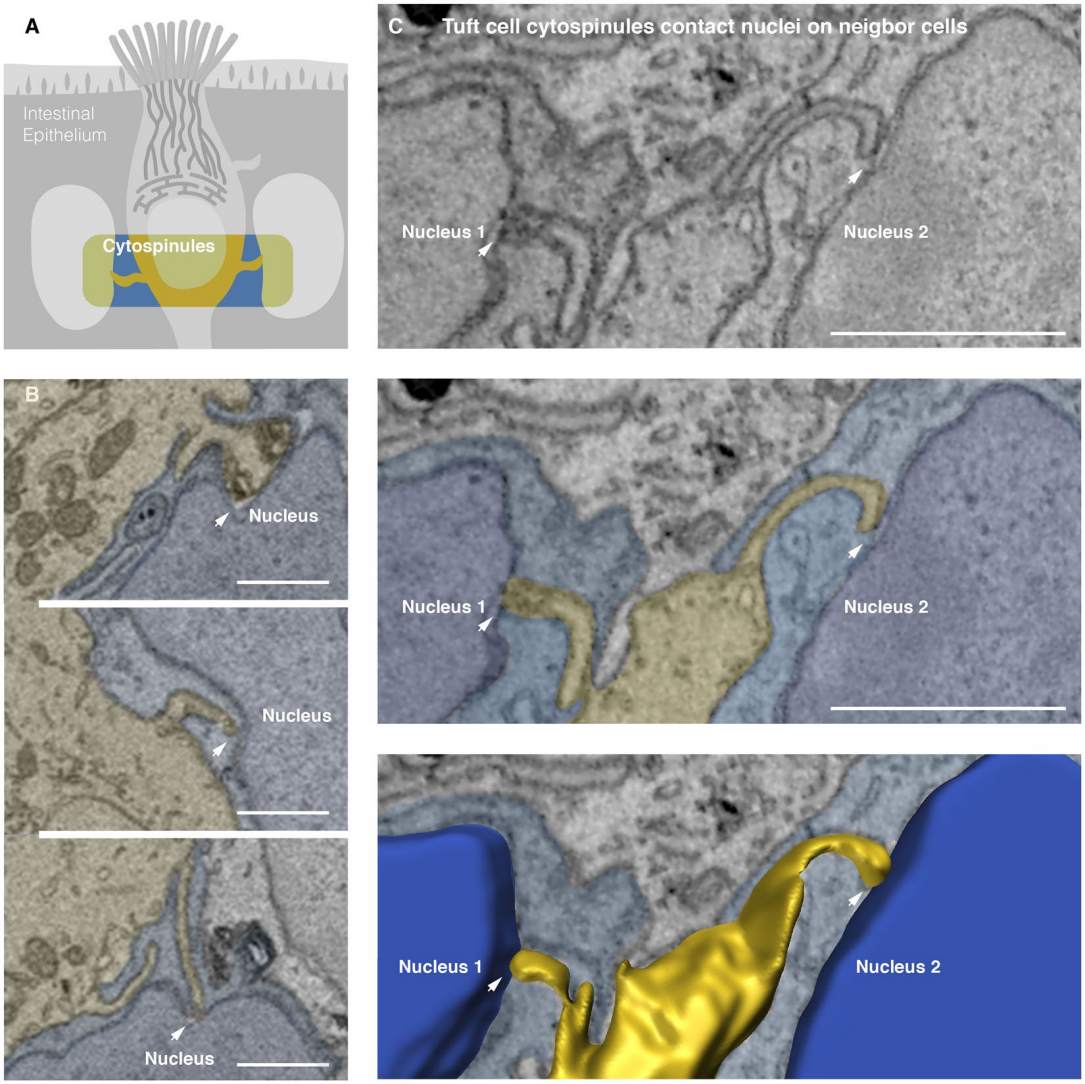
Tuft cell cytospinules contact nuclei of neighbor cells. (**A**) An overview of tuft cell cytospinules. These lateral cytoplasmic projections of tuft cells penetrate the cytoplasm and contact the nucleus of neighboring epithelial cells. (**B**) Examples of cytospinules (arrows) projecting from a tuft cell into the nuclei of neighboring cells. (**C**) Two cytospinules extend from one tuft cell to contact the nuclei of two different epithelial cells. Points of contact are within the dashed yellow circles. The nuclear membrane often invaginates the cytospinule and the point of contact is dark and electron dense. (**D**) Volume rendering of the cells from panel B shows tuft cytospinules contacting the nuclei of adjacent cells. The cytospinules extend for several microns and their full anatomy and connectivity can only be observed by volume rendering. Bars = 1 µm.

Each of the cells analyzed had three to four cytospinules. Luciano and Reale (1979) first noticed these structures in tuft cells of the gall bladder epithelium^10^. They described them as lateral microvilli that penetrated neighboring epithelial cells, though their final target was unclear. By using volumetric electron microscopy, SBEM and ATUM, we were able to determine the destination of these cytospinules. We discovered that each of the tuft cell cytospinules pierces neighboring epithelial cell until making physical contact with the nucleus. The point of contact is dark and electron dense and the nuclear membrane often wraps around the tip of the cytospinule (Fig. 3B,C). Cell-to-cell spinules have been described in neuronal post-synapses^22^; however, cell-to-nucleus spinules have not been reported. In neurons, synaptic spinules appear to facilitate the transfer, via endocytosis, of molecular cargo from the post-synapse of one neuron into the presynapse of a connecting neuron. Genetic material, such as microRNAs, could also be exchanged through cytospinules via exosomes and microvesicles. This mechanism of intercellular communication has been documented in other cells^23–25^. The anatomy and location in tuft cells cytospinules suggest a role for targeting the transfer of molecular cargo to the nuclei of neighboring epithelial cells.

### A molecular passage to the endoplasmic reticulum

**Figure 4:**
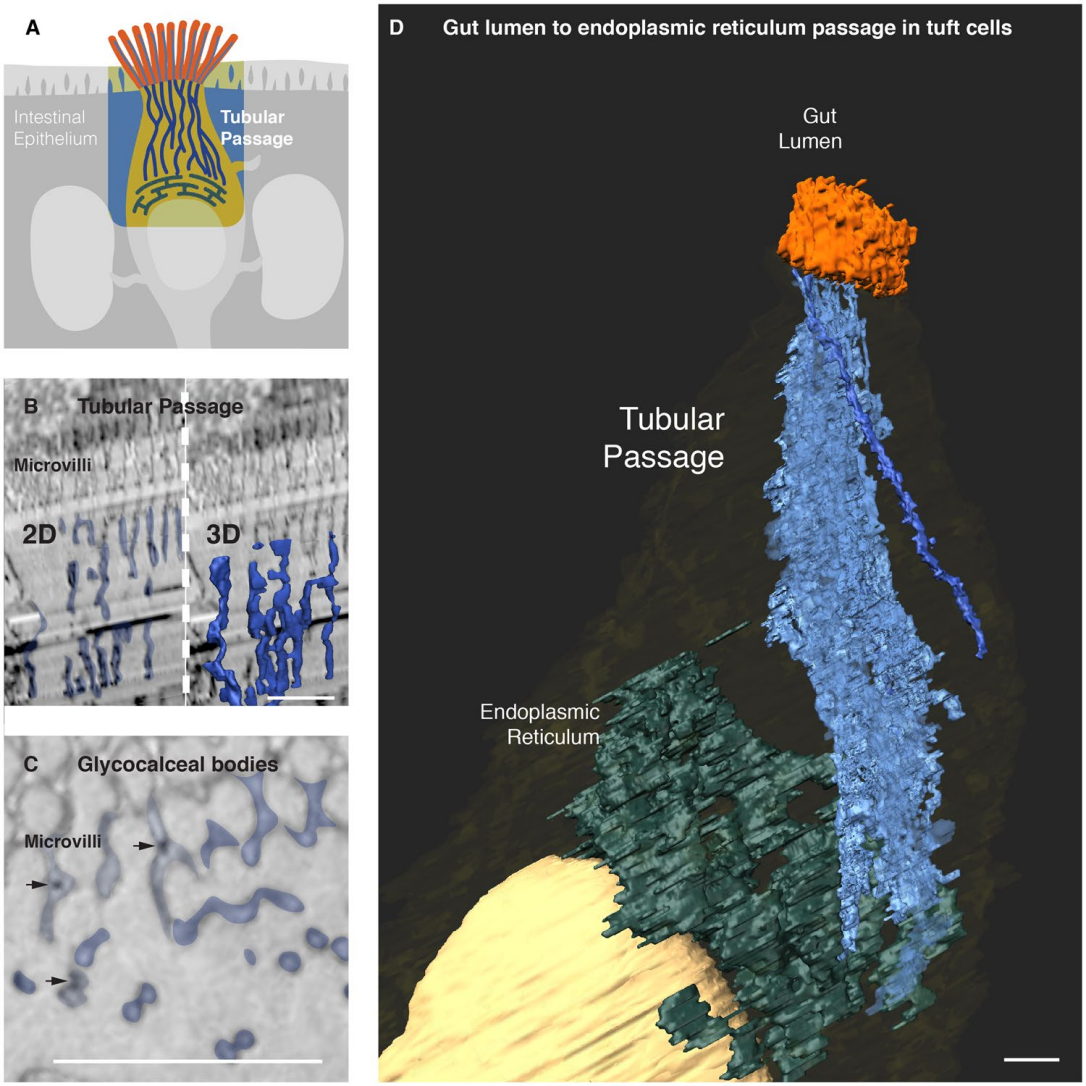
A molecular passage to the endoplasmic reticulum. (**A**) An overview of a passage in the tuft cell connecting its endoplasmic reticulum to the gut lumen. (**B**) A continuous tubular passage starting at the base of the tuft cell microvilli (left) becomes evident after volume rendering of ATUM images (right). (**B**) Glycocalceal bodies (arrows) were observed within microvilli as well as within the tubular passage. (**D**) Volume rendering of ATUM images reveals a passage in tuft cells. It is formed by tubules that start at the base of the microvilli and extend to the endoplasmic reticulum. The tubules appear to merge with the endoplasmic reticulum but this cannot be discerned at the current resolution (4 nm/pixel). See also Video 2. Bars = 1 µm.

Beneath their microvilli, tuft cells have a distinctive array of tubules and vesicles. It has been suggested that these molecular complex may form a tubulovesicular network made of structural proteins such as citokeratin 18, fimbrin, and villin^26^. However, attempts to render the volumetric ultrastructure of the network have lacked sufficient Z resolution to prove the existence of a network^11^. These tubular network was also evident in our SBEM and ATUM images (Fig. 4B). The tubules began at the base of the tuft cell microvilli and extended deep into the tuft cell cytoplasm. The tubules end in the tuft cell endoplasmic reticulum. This molecular passage connects the luminal end of the tuft cell with its endoplasmic reticulum. It is formed by tubules that start at the base of the microvilli and extend to the endoplasmic reticulum. The tubules appear to merge with the endoplasmic reticulum but this cannot be discerned at the current resolution (4 nm/pixel). Such interactions between microtubules and endoplasmic reticulum have been previously described in other cell types^27, 28^. Trapped within the tubular passage, there were glycocalceal bodies (small electron dense spheres) (Fig. 4C). These enigmatic structures are less than 30 nm, electron dense, and often intercalated within microvilli or microtubules. Their relationship with the tubular passage suggests a path for the tuft cell to exchange molecular cargo with the intestinal lumen (Fig. 4D).

The detailed ultrastructural account presented here provides a contextual framework for investigating and understanding the tuft cell intracellular function and its relationships with neighboring cells. Tuft cells mediate neoplastic transformations in colon cancer progression^13, 29^ and orchestrate type II immune responses against parasitic infections^30–32^. The passage from the lumen to the endoplasmic reticulum may serve to facilitate sensing of microbial molecules and secreting cargo into the lumen, whereas the cytospinules represent a potential conduit for targeted delivery of cargo into the nuclei of neighboring epithelial cells (Video 2).

## Methods

### Mice

Animal care and experiments were carried out in accordance with a protocol approved by the Institutional Animal Care and Use Committee of Duke University Medical Center. All mice were bred in-house at the Division of Laboratory Animal Resources of Duke University School of Medicine. The ChAT^BAC^-eGFP and Ai9(RCL-tdT) transgenic lines were acquired from The Jackson Laboratory (Stocks No. 007902 and 007905, respectively). The PyyCRE line was a generous gift of Dr. Andrew Leiter from the University of Massachusetts.

### Tissue collection

Tissue was harvested according to a previously reported protocol^18^. The protocol consists on the following steps: ***Fixation*** *.* Adult mice (8 weeks) were perfused intracardially with cold PBS (0.01 M, 0.9% NaCl) for 1 minute, followed by a PBS solution containing 4% paraformaldehyde and 0.1% glutaraldehyde for 15 minutes. Small tissue sections (3 × 3 mm) were dissected from the distal ileum and proximal colon and incubated in the same fixative at 4 °C for 3 hours. Sections were further trimmed using a Leica VT1000 S vibrating blade microtome (Leica Microsystems Inc., Buffalo Grove, IL) into small blocks of 1000 × 1000 × 50 µm. ***Imaging of tissue blocks with a confocal microscope*** *.* Small blocks were stained with 300 nM DAPI and imaged using a Zeiss 780 inverted confocal microscope. Z-stacks with an optical sectioning interval of 1 µm were obtained. After imaging, blocks containing identified tuft cells (GFP) were post-fixed overnight in 4% paraformaldehyde plus 2.5% glutaraldehyde in PBS. The embedded blocks were stored in PBS at 4 °C until further processing.

### Serial Block Face Scanning Electron Microscopy (SBEM)

Tissues were stained as described by Bohórquez *et al*.^18^. The tissue blocks were infiltrated with resin using the EMbed 812 kit (Electron Microscopy Sciences, Hatfield, PA). To keep the tissue flat, the blocks were embedded between microscope slides, which were cured with liquid releasing agent (Electron Microscopy Sciences, Hatfield, PA). Tissue blocks were then released by pulling the slides apart. Using a dissecting microscope, the orientation of the tissue blocks was aligned to that of the confocal micrographs. Once the regions containing cells of interest had been identified, the tissue block face was manually trimmed to a surface of approximately 500 × 500 µm and mounted on a pin containing CircuitWorks conductive epoxy (ITW Chemtronics, Kennesaw, GA). The block was then cured overnight at 60 °C and coated with colloidal silver liquid (Electron Microscopy Sciences, Hatfield, PA). The block was imaged with a Sigma VP Scanning Electron Microscope (Carl Zeiss Microscopy GmbH, Jena, Germany) equipped with the Gatan 3view system (Gatan Inc., Pleasanton, CA). Low magnification images of the entire block face were acquired, and the tuft cell was identified morphologically from its distinctive microvilli. Serial block face images were acquired in high vacuum mode at a 2.25 kV and 5 nm/pixel (or 15,147X magnification) using 75 nm slices. The resulting serial block face SEM data set contained 500 images in 16-bit.dm3 format. In Fiji, images were next converted to the 8-bit.tiff format and filtered using a 0.8 gaussian blur filter. To minimize the amount of computer RAM memory needed to handle each data set, images were scaled down to 25% of their original size in the x and y dimensions, the z dimension was scaled 1∶2, and the set was saved as.tiff stack. The stack was aligned using the Fiji plugin “linear stack alignment with SIFT” set to translation mode and then cropped using the “crop 3D” plugin in order to focus on the region containing the cell of interest.

### Automated Tape-collecting Ultra Microtome (ATUM) Scanning Electron Microscopy

Tissues were stained according to the protocol of Tapia *et al*.^33^, The blocks were embedded in epoxy resin (Polybed, Polysciences; Warrington, PA). The polymerized blocks were then trimmed for serial section collection with an automated tape-collecting device and an ultra microtome (ATUM)^17^. Serial sections (730 in total) were cut at a thickness of 50 nm and collected on kapton tape. The sections were imaged by black-scattered electron microscopy using a Zeiss Sigma field emission scanning electron microscope^34^. Images were stacked using automatically, using a registration plugin in FIJI software called “Linear Stack Alignment with SIFT”. Regions of interest were determined by measuring the depth of the fluorescent tuft cells in the tissue sample within the confocal z-stacks. Then low-resolution scans of the entire sample were obtained and compared to the confocal data in Fiji software to find the exact cell. Distances from key structural features such as microvilli, goblet cells, or lamina propria were taken into account to correlate structures. Final images of the volume containing the tuft cell were collected for each section at a resolution of 4 nm/pixel and an image size of 12,233 × 12,233 pixels. To minimize the amount of computer RAM memory needed to handle the data set, images were scaled down to 33% of their original size and saved as a tiff stack. The images were aligned using the Linear Stack Alignment with SIFT algorithm from FIJI software.

### Surface Segmentation, volume rendering, and 3D Data Visualization

Segmentation of cellular membranes was performed using Imaris 8.2 (Bitplane, South Windsor, CT) as described by Bohórquez *et al*.^18^. The cell features of interest were reconstructed by manual segmentation of images using the “surfaces” tool. The contour option in drawing mode “distance” was used to trace the contour of the feature of interest in each individual slice where it was visible. Resolution was kept in “auto” mode during surface creation. Images were obtained in Imaris using the snapshot tool at a resolution of 600 dpi and a size of 5000 × 5000 pixels. Videos were produced using the “animation” tool by adding key frames manually and the final recording performed at 24 frames per second.

## Electronic supplementary material

SI Information

Video 1
