# Supplementary material for: The intestinal tuft cell nanostructure in 3D
Source: Sci Rep. 2017 May 10;7:1652. doi: 10.1038/s41598-017-01520-x (PMC5431925; doi:10.1038/s41598-017-01520-x)
Supplement: Supplementary file 1 — SI Information [file 41598_2017_1520_MOESM1_ESM.pdf]

The intestinal tuft cell nanostructure in 3D

Ben Hoover[1‡], Valentina Baena[2‡], Melanie M. Kaelberer[3], Feven Getaneh[1], Skarleth Chinchilla[3], and Diego V. Bohórquez[3, 4*]

Supporting Information

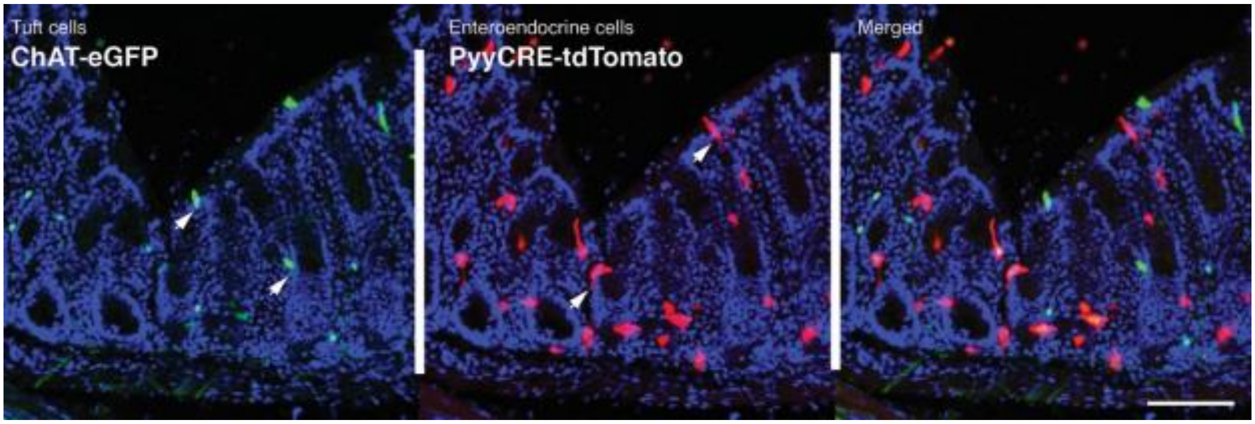

S1 Fig. ChAT-GFP as a marker of tuft cells. In the colon of a triple transgenic animal (ChAT-GFP::PyyCRE::tdTomato), cholinergic nerve fibers and epithelial tuft cells (green-arrows) are clearly distinguhised from Pyy-secreting enteroendocrince cells (red-arrows). Bar = 10 µm

Video 1. The ultrastructure of the intestinal tuft cell in 3D.
